# Oct4/Sox2 Binding Sites Contribute to Maintaining Hypomethylation of the Maternal *Igf2/H19* Imprinting Control Region

**DOI:** 10.1371/journal.pone.0081962

**Published:** 2013-12-06

**Authors:** David L. Zimmerman, Craig S. Boddy, Christopher S. Schoenherr

**Affiliations:** 1 Department of Cell and Developmental Biology, University of Illinois Urbana-Champaign, Urbana, Illinois, United States of America; 2 Biology Department, College of the Ozarks, Point Lookout, Missouri, United States of America; 3 Division of Medical Education, Washington University School of Medicine, Saint Louis, Missouri, United States of America; Universität des Saarlandes, Germany

## Abstract

A central question in genomic imprinting is how parental-specific DNA methylation of imprinting control regions (ICR) is established during gametogenesis and maintained after fertilization. At the imprinted *Igf2/H19* locus, CTCF binding maintains the unmethylated state of the maternal ICR after the blastocyst stage. In addition, evidence from Beckwith-Wiedemann patients and cultured mouse cells suggests that two Sox-Oct binding motifs within the *Igf2/H19* ICR also participate in maintaining hypomethylation of the maternal allele. We found that the Sox and octamer elements from both Sox-Oct motifs were required to drive hypomethylation of integrated transgenes in mouse embryonic carcinoma cells. Oct4 and Sox2 showed cooperative binding to the Sox-Oct motifs, and both were present at the endogenous ICR. Using a mouse with mutations in the Oct4 binding sites, we found that maternally transmitted mutant ICRs acquired partial methylation in somatic tissues, but there was little effect on imprinted expression of *H19* and *Igf2*. A subset of mature oocytes also showed partial methylation of the mutant ICR, which suggested that the Sox-Oct motifs provide some protection from methylation during oogenesis. The Sox-Oct motifs, however, were not required for erasure of paternal methylation in primordial germ cells, which indicated that the oocyte methylation was acquired post-natally. Maternally inherited mutant ICRs were unmethylated in blastocysts, which suggested that at least a portion of the methylation in somatic tissues occurred after implantation. These findings provide evidence that Sox-Oct motifs contribute to ICR hypomethylation in post-implantation embryos and maturing oocytes and link imprinted DNA methylation with key stem cell/germline transcription factors.

## Introduction

Genomic imprinting is an epigenetic phenomenon that employs DNA methylation to direct unequal expression of the two parental alleles of a gene. Genes subject to genomic imprinting are characterized by sequences known as differentially methylated regions (DMRs), which are methylated only on the maternal allele for some imprinted genes, and only on the paternal allele for others. A subset of DMRs are the key elements directing mono-allelic transcription of one or more imprinted genes and are often termed ‘imprinting control regions’ (ICRs). Their parental-specific DNA methylation imprints generally are established during oogenesis or spermatogenesis and are maintained after fertilization in somatic cell lineages. ICRs regulate transcription of imprinted genes by several mechanisms, including methylation-dependent repression, expression of non-coding RNAs, and long-range chromatin interactions [1]. In addition to DNA methylation, certain histone modifications are frequently associated with ICRs, and there is evidence that they also can act as an imprint [2].

An ICR upstream of the *H19* gene on mouse chromosome 7 and on human chromosome 11p15 coordinates the reciprocal expression of *H19* and *Igf2* by controlling access to shared enhancers. Methylation differences between the parental alleles are established in the gametes, as CpGs within the *Igf2/H19* ICR are hypomethylated in oocytes and hypermethylated in sperm. After fertilization, this differential methylation is maintained in essentially all somatic cells. To direct imprinted expression of *Igf2*, the ICR acts as a CTCF-dependent chromatin boundary or insulator that blocks interaction of the *Igf2* promoter with downstream enhancers through the formation of cohesin-dependent intra-chromosomal loops [3]–[5]. Conversely, hypermethylation of the paternal ICR represses *H19* expression and blocks CTCF binding, which allows interaction of the enhancers with the *Igf2* promoter via an alternative loop structure [6]–[8]. Consistent with this model, deletion of the ICR or mutation of the CTCF sites in mice results in biallelic expression of *Igf2* [9]–[11]. The imprinting mechanism appears to be the same in humans, as some patients with Beckwith-Wiedemann Syndrome (BWS) show biallelic expression of *IGF2* that is associated with the inheritance of maternally methylated or deleted ICRs [12].

Although the acquisition of ICR methylation is often considered the main imprinting ‘mark’, maintaining the unmethylated state of ICRs is also part of an active imprint. The acquisition of ectopic methylation by maternal *Igf2/H19* ICRs with CTCF site mutations demonstrates this concept of active maintenance of hypomethylated ICRs [9]-[11], [13], [14]. During embryogenesis, loss of CTCF binding at one or more of the four binding sites results in ICR methylation in somatic cells, and biallelic transcription of *Igf2* [9]–[11], [13], [14]. Conversely, mutations that allow CTCF to bind methylated paternal ICRs have also shown that CTCF can facilitate demethylation of the paternal allele in mouse somatic tissue [15]. However, CTCF binding is not required for erasure of paternal methylation imprints in primordial germ cells (PGCs) or for protection of the ICR from *de novo* methyltransferases during postnatal methylation imprint establishment in oocytes [9]–[11]. In addition to CTCF, the *Igf2/H19* ICR has a conserved pair of Sox-Oct motifs located between CTCF sites 2 and 3 in mice and in both A repeats in humans [16], [17]. Both the mouse and human motifs are comprised of a site for Sox proteins immediately adjacent to an octamer element, which binds POU family proteins. The motifs have been shown to bind Sox2, Oct4 and Oct1 *in vitro* and can drive demethylation of partially methylated ICR transgenes in a mouse embryonic carcinoma cell line [16], [17]. Furthermore, point mutations that disrupt Oct4 binding are associated with abnormal maternal ICR methylation in a small number of BWS patients [18], [19].

In this study, we investigated the role that the octamers within the Sox-Oct motif play in establishing and maintaining the maternal hypomethylation imprint of the mouse ICR. Using a mouse with mutations in the octamers, we found that intact Sox-Oct motifs were required to protect the maternal ICR from *de novo* methylation in somatic tissues and in oocytes, but were not essential for imprint establishment or mono-allelic expression of *H19* and *Igf2*. Taken together, our results suggest that the stem cell/germline factors Sox2 and Oct4 participate in ICR hypomethylation.

## Results

### CTCF and octamer sites regulate ICR DNA methylation in F9 cells

**Figure 1:**
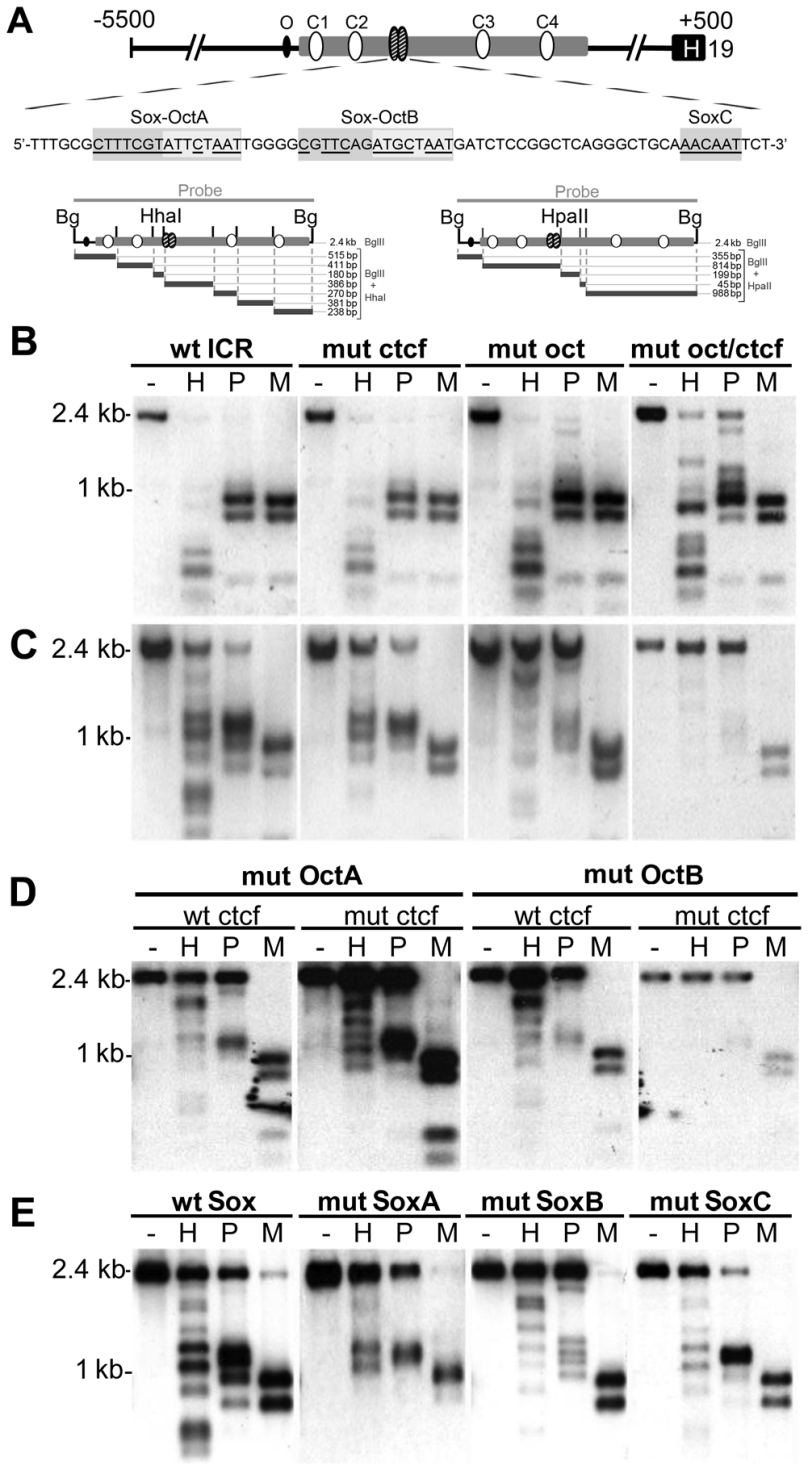
Analysis of ICR transgene methylation in F9 cells. (**A**) Top: Structure of mouse ICR transgene depicting consensus octamer, O (black oval); four CTCF sites, C1–C4 (white ovals); two Sox-Oct motifs (striped oval). Middle: ICR sequence encompassing both Sox-Oct motifs (A and B) and a downstream Sox site (SoxC). Nucleotides matching the respective consensus sequences are underlined. Bottom: *Hha*I, and *Hpa*II restriction maps for the 2.4 kb *Bgl*II,(Bg) ICR fragment. (**B–E**) Southern blot analysis of F9 genomic DNA prepared from polyclonal populations with stably incorporated WT or mutant ICR transgenes. DNA was digested with *Bgl*II followed by digestion with *Hha*I (H), *Hpa*II (P), or *Msp*I (M). The 2.4kb *Bgl*II fragment was used as a probe. The endogenous ICR is visible only as a faint signal compared to that generated by the multicopy transgene. Each blot is representative of the data obtained from three independent polyclonal populations. (**B**) Stable transfectants obtained from unmethylated ICR constructs containing either a WT sequence, (wt ICR); mutations in all four CTCF sites, (mut ctcf); mutation in the three octamer sites, (mut oct); or in all octamer and CTCF sites (mut oct/ctcf). (C–E) All plasmids were *in vitro* methylated at *Hha*I and *Hpa*II sites prior to transfection. (**C**) Stable transfectants obtained from the same WT and mutant constructs as in (B) but with *in vitro* methylation (**D**) Stable transfectants obtained from methylated constructs with OctA or OctB mutations combined with either WT or mutant CTCF sites. (**E**) Stable transfectants obtained from methylated constructs with separate mutations in SoxA, SoxB or SoxC. Both the mutant and WT (wt sox) constructs contain two modified *Spe*I cloning sites flanking the respective Sox-Oct elements. See Table S1 for WT and mutant sequences.

The mouse *Igf2/H19* ICR has four CTCF sites and a closely spaced pair of Sox-Oct motifs (Sox-OctA and Sox-OctB) that lie about 200 bases from CTCF site 2 (Fig. 1A). Previous work indicated that CTCF and octamer sites regulate CpG methylation of the ICR, but whether these sites collaborate is unknown [9]–[11], [13], [16]–[19]. To more fully address octamer functions in the ICR, we searched for similar sequences and found an additional consensus octamer site 450 bp upstream of CTCF site 1 (Fig. 1A). To determine whether the octamers and CTCF sites cooperate in regulating ICR methylation, we mutated the three octamers and four CTCF sites in ICR-containing plasmids and assessed the methylation status of the transgenes after their stable incorporation into F9, C2C12 or 3T3 cells. F9 embryonic carcinoma cells were chosen, as they express Sox2 and Oct4, and have demonstrated both *de novo* methylation and demethylation of transgenes. C2C12 and 3T3 cells, on the other hand, do not express Sox2 and Oct4.

Using methylation sensitive Southern analysis, we found that integrated transgenes containing the wild type ICR and *H19* promoter did not acquire methylation in F9 cells, while mutation of the octamers resulted in a small amount of *de novo* ICR methylation, which is shown by the incomplete digestion of the *Bgl*II fragment with *Hpa*II (Fig. 1A and B). In contrast to results seen in mice, mutation of the four CTCF sites did not lead to increased methylation of the ICR (Fig. 1B). The protective activity of CTCF was apparent only when both the CTCF and octamer sites were mutated, as the double mutant ICR showed less digestion by *Hha*I and *Hpa*II than the octamer mutant alone (Fig. 1B).

In addition to protection from *de novo* methylation, binding by certain transcription factors can drive demethylation of partially methylated sequences. We tested the demethylation activity of the CTCF and octamer sites using stably integrated plasmids that had been treated with *Hha*I and *Hpa*II methyltransferases, and found that both WT and CTCF mutant ICRs were demethylated in F9 cells (Fig. 1C). By contrast, ICR transgenes with mutant octamers retained most of their partial methylation. Moreover, ICR constructs with single mutations in either OctA or OctB retained methylation to the same degree as the double octamer mutant (Fig. 1D). Combining CTCF and octamer mutations resulted in a modest additional decrease in demethylation activity (Fig. 1C and D). Transgenes containing mutations in the distal octamer showed wild type levels of demethylation regardless of CTCF site functionality, suggesting that it is not involved in regulating ICR methylation (Fig. S1A).

**Figure 2:**
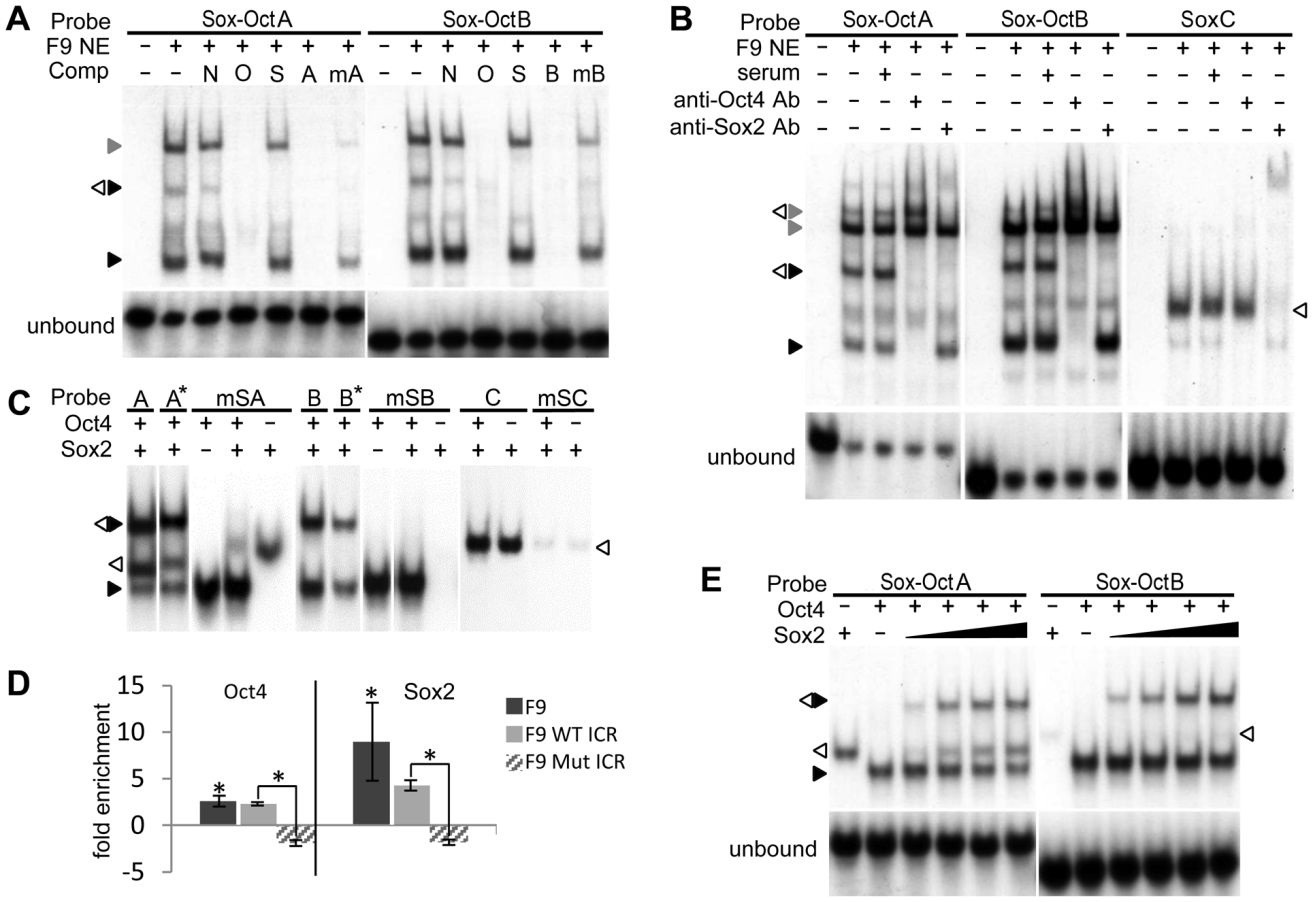
Sox2 and Oct4 binding to the ICR. EMSAs were performed using WT or mutant ICR probes as indicated with either F9 NE (**A, B**) or recombinant Oct4 and Sox2 (**C, D, F**). (**A**) The effect of octamer specific point mutations on Oct4 and Sox2 binding was assessed by addition of nonspecific (N) and specific unlabeled DNA competitors - oct (O), sox (S), ICR Sox-OctA and B (A and B), or mutant ICR Sox-OctA and B (mA and mB). (**B**) Oct4 and Sox2 supershift assays. Sox2 and Oct4 protein-DNA complexes (white and black triangles respectively) were identified by their respective antibodies (Ab). Presumptive Oct1 complexes are indicated by a grey triangle. (**C**) Recombinant Sox2 binding to Sox-OctA, B and SoxC. ICR probes with mutations to the Sox elements (mSA, mSB, and mSC) were used to confirm Sox2 binding sites. (**D**) Methylation-independent binding of Oct4 and Sox2 to the ICR Sox-Oct motifs Wild type ICR Sox-OctA and B probes (A* and B*) were methylated at all CpGs. (**E**) Oct4 and Sox2 ChIP assays. Enrichment of endogenous F9 or WT and mutant transgenic ICR octamers by Oct4 and Sox2 ChIP was determined by qRT-PCR and normalized to serum controls. Error bars represent standard deviation of the mean (n = 3). Statistical significance of Oct4 and Sox2 enrichments was shown using Student's t-test (*p<0.05). (**F**) Cooperative binding of Sox2 to Oct4 complexes. Cooperative binding characteristics of Sox2 to the ICR were assessed by measuring the amount of binary and ternary protein-DNA complexes formed using a fixed amount of Oct4 and increasing amounts of Sox2. See Table S1 for a complete list of probe and competitor sequences.

Octamer mediated demethylation of the ICR was both specific to embryonic carcinoma cells and limited to ICR constructs with low density methylation. Partially methylated WT and mutant ICR transgenes stably integrated into either mouse C2C12 or NIH3T3 cells remained methylated (Fig. S1B), which is consistent with a requirement for Sox2 and Oct4 for demethylation. In addition, these results suggest that Oct1, which is expressed in F9, C2C12 and NIH3T3 cells and can bind the ICR octamers (Fig. 2A, 2B and data not shown), is not sufficient to drive ICR demethylation. In contrast to partially methylated plasmids, WT ICR transgenes fully methylated *in vitro* with *Sss*I methyltransferase remained highly methylated in F9 cells (Supplementary Material, Fig. S1C). Thus, the presence Sox2/Oct4 is not sufficient to drive demethylation of a completely methylated ICR in F9 cells.

### Sox binding sites regulate ICR methylation in F9 cells

Octamers are often found adjacent to Sox sites, with binding of both Sox and POU factors required to form a functional unit. We tested the importance of the Sox binding sites in the Sox-Oct motifs using the F9 demethylation assay and partially methylated ICR constructs containing mutations that disrupted each Sox site (Fig. 1E). The Sox site mutations prevented formation of a Sox2-Oct4 ternary complex but did not alter Oct4 binding to the adjacent octamer (Fig. 2C). Southern analysis demonstrated a modest reduction in ICR demethylation activity when SoxA was mutated. However, mutation of the SoxB exhibited a greater loss in demethylation activity, which was comparable to the single octamer mutations. We also identified a sequence (SoxC) 22 bps downstream from Sox-OctB that bound Sox2 *in vitro* (Fig. 1A and 2B). Mutation of this element, however, did not affect ICR demethylation alone or in combination with the Sox-OctA mutant (Fig. 1E and Fig. S1D).

### Oct4 and Sox2 bind cooperatively to the ICR Sox-Oct motifs

The limited demethylation of WT transgenes in NIH3T3 and C2C12 cells suggested that Sox2 and Oct4 binding mediates ICR demethylation. Consistent with this idea, chromatin immunoprecipitations (ChIP) from F9 cells showed Oct4 and Sox2 enrichment at both the endogenous and wild type transgenic ICRs, but not at the octamer mutant ICR (Fig. 2D). Analysis of published data from genome-wide Oct4 and Sox2 ChIPs also revealed that the pluripotency factors are localized to the Sox-Oct motifs in mouse embryonic stem (ES) cells (data not shown) [20]. Furthermore, EMSAs performed with F9 nuclear extract showed that both motifs formed heterodimeric complexes with Oct4 and Sox2, and the same octamer mutations that disrupted ICR demethylation also prevented Oct4 binding *in vitro* (Fig. 2A and 2B). A lower mobility complex, which is likely to contain the POU factor Oct1, also was present in EMSAs with F9 nuclear extract (Fig 2A). Thus, Oct1 or other POU factors could contribute to or inhibit ICR demethylation. Because the Sox-Oct-dependent demethylation in F9 cells was limited to partially methylated transgenes, we performed EMSAs with ICR probes methylated at all CpGs and found that the methylation did not inhibit formation of Sox2/Oct4 complexes (Fig. 2C). SoxC, which lacked demethylation activity, produced a clear complex with Sox2 but was not able to form a Sox2-Oct4 complex (Fig. 2B and C).

To assess the potential cooperative binding characteristics of Oct4 and Sox2 to the Sox-Oct motifs, we performed EMSAs with recombinant proteins and found that Oct4 bound to both elements equally well (Fig. 2C and E). Sox2, however, bound weakly to the WT and Sox site mutant Sox-OctB probes, and bound to the WT and Sox site mutant Sox-OctA probes almost equally well (Fig 2C and E). Sox2's binding to the mutant Sox-OctA probe is likely to be through the octamer, as it contains a Sox consensus site (data not shown). Although the ectopic binding by Sox2 complicated our ability to measure it accurately, we were able to demonstrate cooperative binding between Sox2 and the Oct4-Sox-OctA complex. First, we established EMSA conditions in which no more than 10–20% of the Sox-OctA probe was bound by either Sox2 or Oct4 when added separately. If the two factors bound independently, then only 1–4% of the probe would be expected to be in a ternary complex with Sox2 and Oct4. However, we found roughly equal amounts of binary and ternary complexes (Fig. 2E), which when quantified indicated a 6–10 fold enhanced binding of Sox2 to the Oct4-DNA complex (Supplementary Material, Fig. S2aA). For the Sox-OctB motif, the amount of ternary Sox-OctB complex was at least 50 fold more than that expected from independent binding of Oct4 and Sox2 (Fig. S2A). In this case, the cooperativity was much more pronounced as Sox2 alone bound to less than 1% of the probe (Fig. 2E). Conserved Sox-Oct motifs in the Human ICR A2 repeat also demonstrated cooperative binding of recombinant Sox2 and Oct4 in EMSAs (Fig. S2B).

Given the proximity of the two Sox-Oct motifs to one another, it was unclear if Sox2 would be occluded from binding between the two Oct4 proteins. Using probes containing both Sox-Oct motifs, we found that recombinant Oct4 and Sox2 were capable of forming several complexes with the probe, including one that was likely to represent full occupancy of both motifs (Fig. S2C). However, this complex represented a small fraction of observed complexes, making it difficult to confidently assign its composition. To simplify the EMSAs, we used a human ICR A2 probe with a mutant Sox site upstream of the first octamer. This probe showed the formation of an Oct4-Sox2-Oct4 complex, which indicated that there is sufficient space for Sox2 to bind between the two Oct4 molecules (Fig. S2C).

### The ICR Sox-Oct motifs activate transcription in F9 cells

**Figure 3:**
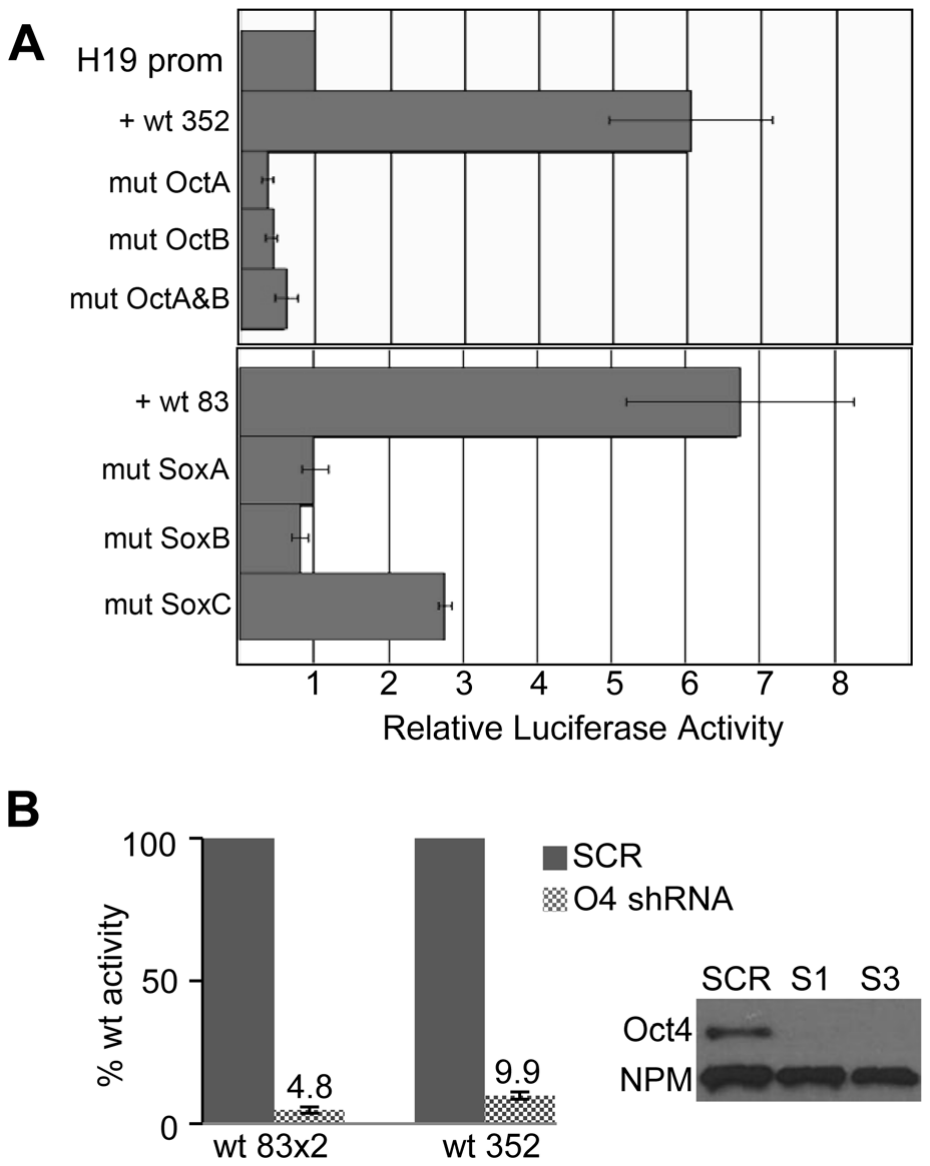
ICR Sox-Oct motifs activate transcription in F9 cells. (**A**) F9 cells were co-transfected with a β-galactosidase reporter for normalization and an unmethylated luciferase reporter construct driven by the H19 promoter alone or in combination with either a 352 bp or an 83 bp ICR fragment which included WT or mutant Sox-Oct motifs without CTCF sites. Normalized luciferase activities are shown relative to the activity of the H19 promoter alone. (**B**) The effect of Oct4 shRNA knock-down on relative luciferase activity is shown for constructs containing either two copies of the wt 83 bp ICR fragment (wt 83×2) or the wt 352 bp fragment. S1 and S3 indicate two different Oct4 shRNAs. SCR indicates scrambled control shRNA. ‘O4 shRNA’ includes both S1 and S3 shRNA knockdown samples. Western blot of F9 cell extract (CE) from S1, S3 or SCR treated cells probed with α-Oct-4 and α-NPM antibodies.

The capacity of Oct4 and Sox2 to initiate DNA demethylation along with transcriptional activation during cellular reprogramming has been well established [21]. A similar correlation between the ability to activate transcription and to drive DNA demethylation has been demonstrated for other transcription factors, suggesting a mechanistic link between the two events.To determine whether transcriptional activation by the ICR Sox-Oct motif correlated with the demethylation seen in the stable transfections, we placed ICR fragments containing the elements upstream of a luciferase gene driven by the H19 promoter (Fig. 3A). In transiently transfected F9 cells, the Sox-Oct regions activated the reporters more than five-fold compared to the H19 promoter alone. Mutation of one or both octamers abolished transactivation and produced a modest repression. Mutations disrupting the SoxA and B binding sites also abolished transcriptional activation. Mutating SoxC lead to a modest reduction in luciferase expression, suggesting that this element is functional. To determine if Oct4 was required for transactivation, we transfected F9 cells that had been depleted of Oct4 by shRNAs and found that relative luciferase expression dropped at least 90% compared to control shRNA expressing cells (Fig. 3B). Consistent with the Oct4 knock-down, mouse 3T3 cells lacking endogenous Oct4 and Sox2 expression were transiently transfected with ICR-reporter constructs and found to have luciferase levels equal to or less than that of H19 promoter controls (data not shown). Therefore, both transcriptional activation in transient assays and DNA demethylation of integrated ICR transgenes appear to share similar requirements for cooperative Oct4 and Sox2 binding to both Sox-Oct motifs.

### Octamers are required for maintenance of the unmethylated maternal ICR in somatic cells

**Figure 4:**
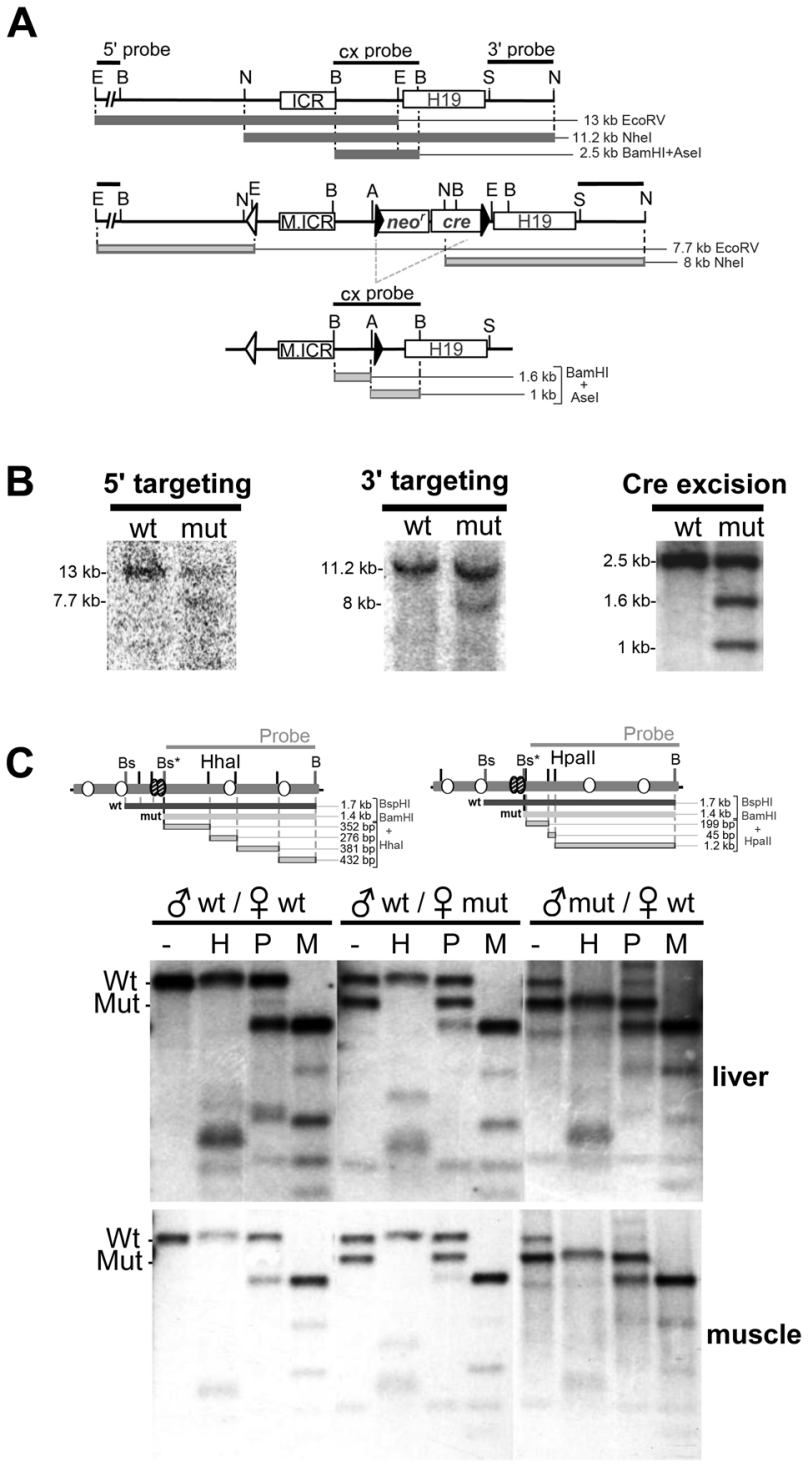
Octamer mutations increase maternal ICR methylation in neonatal somatic tissues. **(A)** Restriction map for the *H19/Igf2* locus, targeting vector insertion and the targeted locus following excision of the selection cassette. The neomycin resistance (*neo^r^*) and Cre recombinase (*cre*) genes were removed in the germline of male chimeras by testes-specific expression of Cre. Restriction sites are *Ase*I (A), *BamH*I (B), *Ecor*V (E), *Nde*I (N), and *Sal*I (S). **(B)** Southern blot analysis to confirm targeting of the mutant ICR (M.ICR) was carried out on *Ecor*V or *Nde*I digested WT and targeted ES cell DNA hybridized to 5′ and 3′ probes, respectively. Correct Cre-mediated excision of the selection cassette was confirmed by *BamH*I and *Ase*I-digestion of tail DNA from F1 mice. **(C)** Methylation sensitive Southern blot analysis was performed on DNA from neonatal liver and muscle from WT and heterozygous mutant mice. Genomic DNA was digested with *BamH*I and *BspH*I (Bs) and then *Hha*I (H), *Hpa*II (P), or *Msp*I (M). Restriction maps of ICR are shown. The mutation in OctB created an additional *BspH*I (Bs*) restriction site. The 1.4 kb mutant ICR *BspH*I-*BamH*I fragment was used for the probe. The results shown are representative of methylation sensitive Southern analysis performed on six pups from four different mutant females.

To determine if the octamers participated in establishing or maintaining the ICR's differentially methylated state *in vivo*, we used homologous recombination and ES cells to create mice with mutations in the three octamer sites (Fig. 4A and B). Southern analysis of DNA from neonatal liver and muscle (Fig. 4C) showed that the loss of octamer binding had no effect on the methylation of paternally transmitted mutant ICRs. However, methylation of maternally transmitted mutant ICRs increased significantly, as indicated by reduced *Hpa*II and *Hha*I digestion compared to wild-type maternal ICR DNA. The asymmetric distribution of methyl-sensitive restriction sites in the probed region (with *Hpa*II sites being more 5′ and *Hha*I more 3′ to the octamers) also provided a means for assessing the relative location of methylated CpG downstream of Sox-OctB. Analysis of the banding patterns from *Hpa*II and *Hha*I digests suggests that the majority of mutant allele methylation was concentrated near the octamers while the region including the promoter proximal CTCF sites was largely unmethylated.

**Figure 5:**
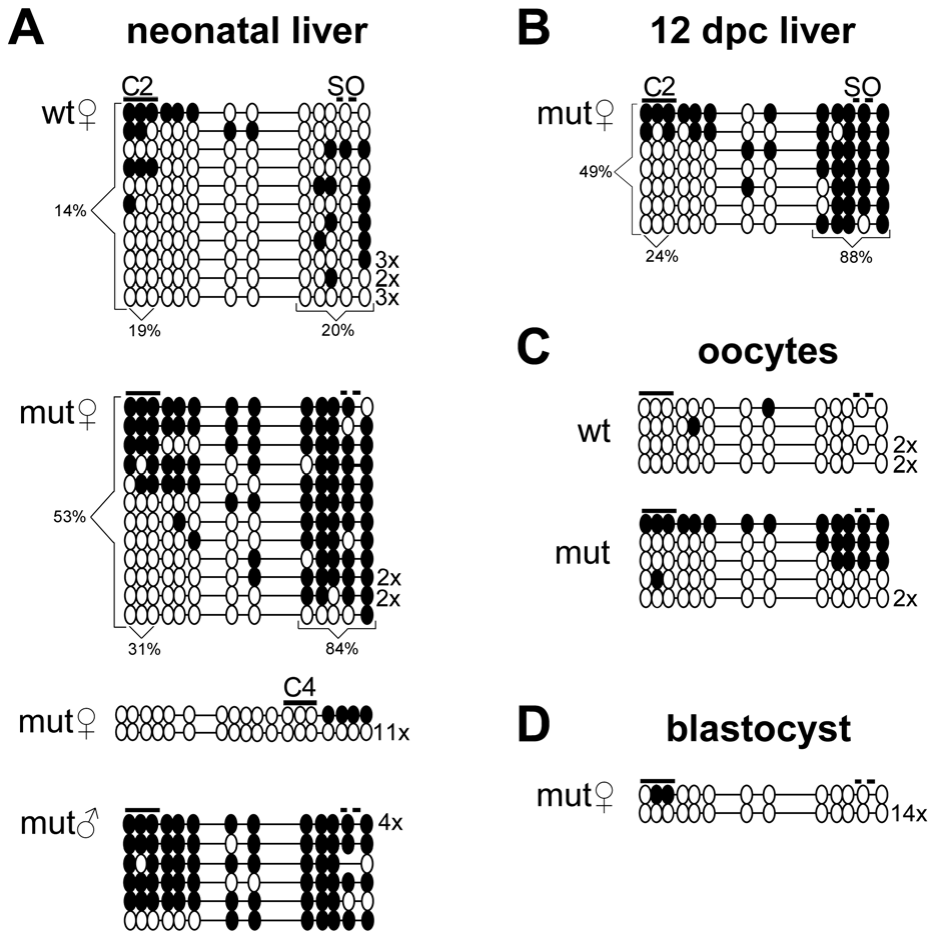
Methylation profiles of WT and mutant ICRs. Each horizontal line of circles represents a unique sequence. Unmethylated and methylated CpGs are presented as open and closed circles, respectively. Multiples of the same methylation pattern are indicated to the right of each sequence. Lines above the circles indicate relative location of CTCF sites 2 and 4 (C2, C4) and the Sox-Oct motifs (SO). The mutant status and the allelic parent of origin are indicated to the upper left of each block of sequences. **(A)** Bisulfite analysis was performed on neonatal liver from three heterozygous mutant mice and **(B)** the maternally transmitted mutant ICRs from one 12 dpc embryonic liver. **(C)** Both WT and mutant bisulfite PCR products from oocytes were the result of four separate reactions each using the DNA from 10–20 oocytes. Oocytes were collected from a combination of homozygous and heterozygous mutant and WT females after superovulation. Each PCR product was cloned separately, and 3–6 clones of each were sequenced. Identical sequences from each product were counted only once. **(D)** Bisulfite analysis of maternally derived mutant alleles from heterozygous blastocysts was the result of two separate reactions of 6–10 blastocysts each. PCR products from each reaction were cloned separately.

To more accurately characterize the increased methylation, we performed bisulfite sequencing of the region including CTCF site 2 and the octamers on DNA from neonatal liver and found increased CpG methylation flanking the mutant Sox-Oct motifs consistent with the Southern analysis. On average, CpGs within 35 bps of the Sox-Oct motifs from maternally inherited mutant alleles were 84% methylated and more than 53% methylated for all CpGs in the fragment. In WT ICRs, CpGs within 35 bps of the Sox-Oct motifs were 20% methylated compared to a 14% average methylation for all CpGs in the amplified fragment (Fig. 5A). In contrast, the three CpGs within CTCF site 2 showed a modest 31% average methylation for the mutant alleles compared to a 19% average methylation for the maternal wild-type alleles. A comparison of WT and mutant maternal alleles from neonatal muscle yielded similar results (Fig. S3). Additional bisulfite analysis of the ICR region surrounding CTCF site 4 from neonatal liver showed virtually no methylation of maternal mutant alleles (Fig. 5A). Paternal methylation imprints were likewise unaffected by the octamer mutations (Fig. 5A and data not shown).

### Octamer mutations increase methylation in oocytes but not blastocysts

To determine if the ectopic methylation of the maternal mutant ICR occurred during oogenesis or after fertilization, we conducted additional methylation analysis of mutant embryos, oocytes and blastocysts (Fig. 5B–D). In somatic tissue from 12 dpc embryos, methylation of the mutant maternal ICR appeared relatively unchanged from methylation patterns present in neonatal tissues (Fig. 5B). In oocytes, bisulfite sequencing of the octamer region revealed that roughly half of the mutant ICRs exhibited methylation patterns similar to those obtained from somatic tissue, while the other half remained unmethylated (Fig. 5C). However, we found the octamer region from mutant blastocysts to be almost completely unmethylated (Fig. 5D). Although we cannot exclude maintenance of the oocyte-derived methylation in some animals, its absence in blastocysts suggests that maternal ICR methylation found in later stages occurred after implantation.

### The octamers are not required for establishing an unmethylated ICR in the primordial germline

**Figure 6:**
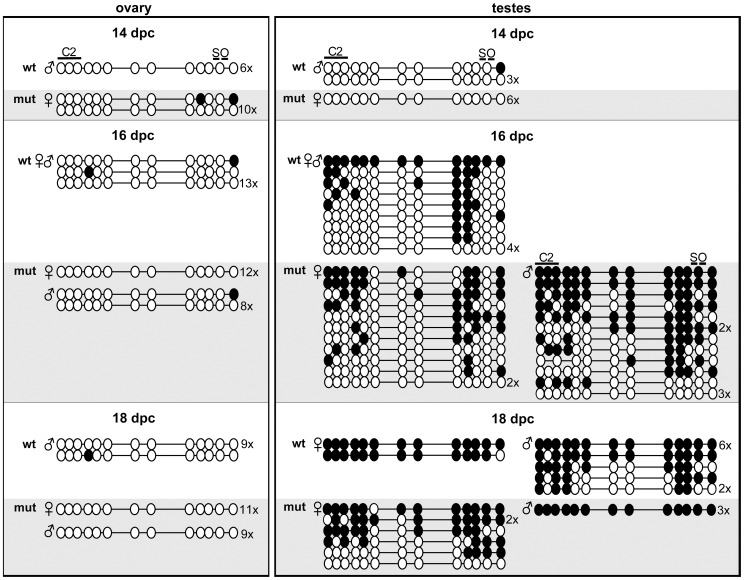
Methylation profiles of WT and mutant ICRs from female and male embryonic germ cells. Bisulfite sequencing of the CTCF site 2 and Sox-OctA and B region are shown for germ cells isolated from 14, 16, and 18 dpc ovaries and testes from heterozygous mutant embryos. WT and mutant alleles are denoted by white and grey backgrounds respectively. The parental origin of alleles is indicated by the symbol to the left of sequence graphical representations. Each block of bisulfite sequences was the result of 1–2 separate PCR reactions performed with DNA from 1×10^3^–1×10^4^ GFP-sorted germ cells obtained from 4–16 pooled gonads. DNAs from separate PCR reactions were cloned separately.

We assessed mutant ICR methylation in germ cells during different stages of embryogenesis to determine if the ectopic methylation in oocytes resulted from a failure to erase paternal methylation imprints or from inadequate protection from *de novo* methylation during normal imprint establishment in oocytes [22], [23]. Primordial germ cells (PGCs) were isolated from 14, 16 and 18 dpc ovaries and testes by crossing ICR mutants with a Pou5f1-EGFP strain and FACS sorting the respective dissociated tissues. 94–99% of GFP+ FACS isolated cells tested also stained positive for alkaline phosphatase (data not shown). Bisulfite analysis of PGCs from 14 dpc ovary and testes showed normal hypomethylation of maternally inherited mutant ICRs (Fig. 6). The ICR octamers also were not required for erasure of paternal methylation in female PGCs as evidenced by the absence of methylation on paternally derived mutant alleles from 16 dpc ovaries. Moreover, no *de novo* methylation of the mutant octamer region was seen in the female germline up to and including 18 dpc development. In embryonic testes, mutant alleles acquired paternal specific methylation imprints irrespective of parental origin (Fig. 6).

### The ICR octamers have little influence on mono-allelic expression of H19 and Igf2

**Figure 7:**
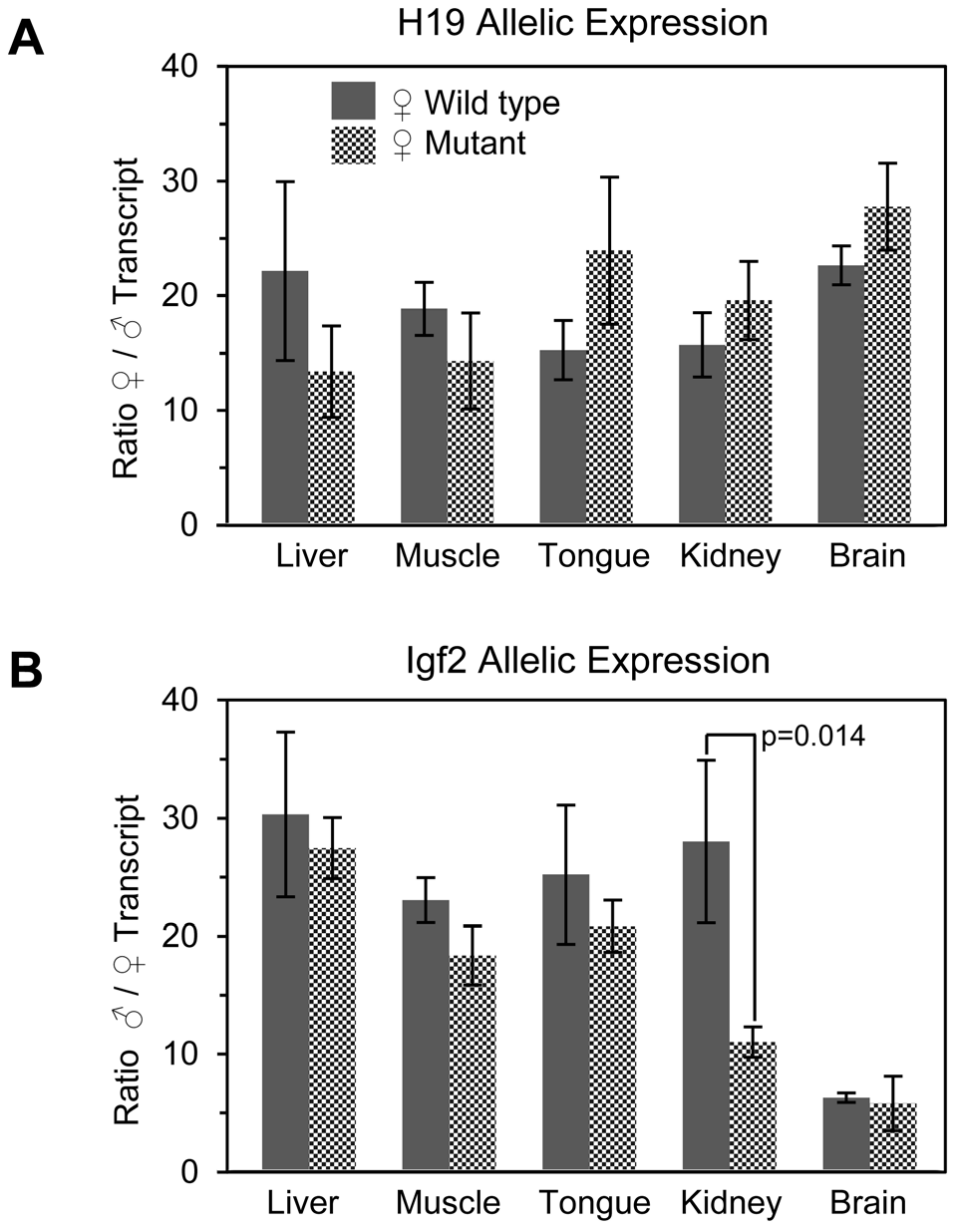
Allelic H19 and Igf2 mRNA expression ratios in neonatal mice. SNuPE analysis was carried out on **(A)** H19 and **(B)** Igf2 cDNA from 4 dpn heterozygous mice having a paternally inherited WT *M. cast*. allele and a maternally inherited WT or mutant B6 allele. The ratio of transcript for each tissue was determined by averaging the individual allelic ratios for 3–5 different mice. Error bars represent the standard deviation of the mean. The p values were generated using Student's t-test.

We performed a single nucleotide primer extension assay (SNuPE) in order to determine if the methylation observed on the maternal mutant ICR was sufficient to alter the normal allelic expression ratios of *H19* and *Igf2* in liver, muscle, tongue, kidney, and brain from neonatal wild type and heterozygous mutant mice. Despite the partial methylation of mutant ICRs in liver and muscle, no significant difference in the ratio of maternal/paternal *H19* allelic expression was observed for the maternally transmitted mutant alleles (Fig. 7A). The ratio of paternal to maternal *Igf2* expression was similarly unchanged in all tissues assayed except for kidney where we observed an apparent 2.5 fold increase in maternal *Igf2* expression (Fig. 7B). We observed no difference in size between neonatal mice that inherited the mutant maternal allele and their WT littermates, which was consistent with normal repression of maternal *Igf2* in most tissues (data not shown).

## Discussion

Genomic imprinting requires establishing and maintaining DNA methylation on one ICR allele and the absence of methylation on the other. At the *Igf2/H19* locus, CTCF binding maintains the maternal ICR in its unmethylated state, but only after fertilization [9]–[11]. However, it is unknown if other DNA binding factors cooperate with CTCF in the early embryo or if germline factors establish hypomethylation in oocytes. We found that Oct4 and Sox2 bind cooperatively to the two Sox-Oct motifs and that both the Sox and Oct site within each motif are required for robust ICR demethylation in F9 cells, suggesting they comprise a single functional unit capable of driving DNA demethylation. Hori et al. showed a similar requirement for both Sox-Oct elements to drive ICR demethylation in P19 cells [17]. We made mice with mutations in the Oct4 elements and found increased maternal ICR methylation after the blastocyst stage and minor methylation increases in oocytes. Thus, the Sox-Oct motifs participate in both establishing a hypomethylated ICR in oocytes and maintaining this state after fertilization. This activity is most pronounced in the maintenance phase and is likely to prevent methylation during the wave of genome-wide remethylation that occurs after implantation [24]. However, unlike the CTCF site mutations, the extent of ectopic methylation was insufficient to cause loss of *Igf2* imprinting, indicating that CTCF plays a greater role than Oct4/Sox2 in maintaining ICR hypomethylation in somatic cells.

Although these results suggest that Oct4/Sox2 and CTCF cooperate to maintain full ICR demethylation, the activities of each factor differed when examined in mice and F9 cells. In mice, the ectopic methylation elicited by the Oct4 site mutations largely centered on the Sox-Oct motif but spread to a lesser degree into CTCF site 2. Moreover, there was no increase in methylation near CTCF sites 3 and 4. This limited spreading of methylation might indicate that protection by Oct4/Sox2 is due to a steric hindrance mechanism or that CTCF limits methylation spreading. In F9 cells, however, the Sox-Oct motif drove demethylation throughout the ICR, which suggests that Oct4 and Sox2 can actively direct DNA demethylation using a mechanism that goes beyond simple steric hindrance and can protect the entire ICR. With CTCF, we found the reciprocal. In F9 cells, the CTCF sites provided little demethylation or methylation protection activity without the Sox-Oct motifs. In mice, however, maternal transmission of an ICR with four mutant CTCF sites resulted in substantial methylation across the ICR [9]. Moreover, CTCF drove substantial demethylation over the entire paternal ICR in mice with mutations that allow CTCF to bind its sites even when methylated [15]. For this mutant ICR, it is important to note that the CTCF site-dependent demethylation may require cooperation with the Sox-Oct motifs, and only local demethylation might be seen if they were also mutated.

There are many possible explanations for the differences between the local and long-range activity of these factors in mice and F9 cells, especially given the complicated nature of active and passive demethylation and *de novo* versus maintenance methylation. They could simply reflect the mechanistic differences between demethylation and blocking methylation in a multicopy transgene array in F9 cells and at the endogenous locus in the mouse. Alternatively, differences in the transcriptional activity of Oct4/Sox2 and CTCF could explain the discrepancies. Transcriptional activation is known to correlate with DNA demethylation, and strong activators such as VP16 can induce greater long-range demethylation than weaker ones [25]. We found a similar correlation with long-range demethylation and transcriptional activation by the Sox-Oct motifs in F9 cells. CTCF can activate transcription [26], but only weakly, and it may be insufficient to target long-range demethylation in F9 cells. In mice, however, CTCF may recruit factors that are not active in cultured cells. Whether the protection or demethylation activity of either of these factors involves recruiting demethylases or blocking DNA methyltransferases remains to be determined.

In contrast to our results with octamer mutations, mice possessing a ∼0.9 kb ICR deletion between CTCF sites 2 and 3, which removes the Oct-Sox pair, did not show ectopic methylation of maternally inherited mutant ICRs [27]. The discrepancy between the point mutation and deletion alleles suggests that the spread of methylation into CTCF site 2 that we detected requires the sequence surrounding the Sox-Oct element. Thus, this region could contain binding sites for methylation nucleating factors, such as Zfp57 or other repressors [28]. If this region has methylation nucleating activity, then the Sox-Oct sites could counter the effects of these putative repressors. In this model, deleting this region would be neutral, as both methylation and demethylation activities would be removed. Consistent with potential repressor activity, we observed that luciferase reporters containing octamer mutations in the context of a 352 bp ICR fragment averaged a near 2-fold reduction in expression compared to the H19 promoter alone (Fig. 3A). In female mice, resetting the *Igf2/H19* ICR imprints requires removing methylation from the paternal ICR during a genome-wide demethylation event in PGCs. We supposed that this demethylation, at least in part, could be directed to the ICR by the Oct4/Sox2 (or related factors) present in PGCs. However we did not see any defects in demethylation in our mutant, indicating that the Sox-Oct motif is not required for paternal imprint erasure. Moreover, the absence of any erasure defects in both the octamer and CTCF site mutants [9], [10] suggests that the ICR demethylation process in PGCs is largely sequence independent.

After global demethylation in PGCs, genome-wide remethylation begins at about 16 dpc in the germline of males. In oocytes, remethylation begins a few days after birth and continues during maturation [22]. In males, methylation of the *Igf2/H19* ICR seemed unperturbed by the octamer mutations, whereas in females the octamer motifs block *de novo* methylation of the Sox-Oct motifs in a subset of mature oocytes. The ectopic methylation in mature oocytes suggests that Oct4/Sox2 or related factors might protect the *Igf2/H19* ICR from Dnmt3L-dependent activity that leads to methylation of ICRs from maternally imprinted genes [22]. Whether this methylation is sufficient to constitute a stable imprint remains to be determined. The absence of ICR methylation in the pooled blastocysts, however, is consistent with the loss of oocyte-derived methylation during the post-fertilization genome-wide demethylation.

The incomplete methylation of the mutant maternal ICRs in both somatic and germline cells indicates that additional factors cooperate with Oct4/Sox2. While it seems clear that CTCF and Sox-Oct sites cooperate to protect the ICR in somatic cells, the evidence for this activity in oocytes is somewhat equivocal. On one hand, mutation of the CTCF binding sites does not lead to ICR methylation in oocytes [9]–[11], while partial ICR methylation was seen in oocytes depleted of CTCF [13]. In any case, cooperation in the germline could be addressed with a double CTCF site and octamer mutant ICR. In this regard, we found that in F9 cells CTCF's methylation protection activity was best demonstrated in conjunction with the octamer mutations.

In five different cases of BWS, point mutations or a deletion affecting one of the octamers in the human ICR has been implicated in loss of *IGF2* imprinting due to ectopic maternal ICR methylation [18], [19]. The mouse model presented here provides direct evidence that maternal transmission of octamer mutations alone can result in partial methylation of the maternal *Igf2/H19* ICR. However, in contrast to the BWS cases, the increase in ICR methylation induced by the mutant octamer was mostly absent from the CTCF sites of maternally inherited alleles, indicating that most sites remained occupied by CTCF. This conclusion is consistent with our finding that imprinted expression of *H19* and *Igf2* was largely unaffected. The differences between the effects in mice and humans suggest that the control of methylation by the conserved octamers may depend on non-conserved aspects of ICR architecture or species-specific differences in DNA methylation machinery. The existence of species-specific requirements for methylation maintenance is suggested by the inability of paternally inherited human ICR transgenes to maintain methylation that was acquired during spermatogenesis [29]. Alternatively, octamer mutations may not be fully penetrant for substantial ICR methylation in humans. Thus, the octamer-dependent BWS cases could represent mutant individuals that were also subject to a stochastic methylation event, or that have genetic polymorphisms that increase DNA methylation.

Protecting hypomethylation imprints of ICRs may be a common function of Oct4 and Sox2 at imprinted loci. For example, the normally hypomethylated human Angelman Syndrome – imprinting center (AS-IC) acquires methylation in mouse oocytes when mutations are made in one of two octamers or an independent Sox site [30]. Similar to the mutant *H19/Igf2* ICR, methylation in oocytes occurs on only a fraction of mutant alleles and is lost during fertilization. The AS-IC also has binding sites for other factors that regulate its methylation [30], which parallels the partial redundancy between CTCF and Oct4/Sox2 at the ICR. In addition to these two loci, we analyzed published genome-wide ChIP data and found that Sox2 and Oct4 also occupied several ICRs from other imprinted loci in ES cells (data not shown). Taken together, these results suggest that Oct4 and Sox2 (or related factors) may collaborate with other transcription factors to establish ICR hypomethylation at several imprinted loci during gametogenesis and then maintain that hypomethylation after blastocyst implantation. Although their function needs to be addressed further, the possibility that factors key to germline and stem cell development participate in setting and maintaining imprints reveals an intimate connection between these processes and could reflect important events in the evolution of genomic imprinting.

## Methods

### Plasmid construction

Mutations in the ICR were created using overlapping PCR with mutant primers. Substitutions at OctB and the upstream octamer (−3248 and −4338 with respect to the H19 start site) created novel *BspH*I sites. The 1.5 kb mutant PCR products were ligated into the *Xho*I and *BspE*I sites of a pcDNA3-luciferase vector containing the mouse H19 upstream region (from −5547 to +695) with either WT or mutant CTCF sites. WT and mutant Sox constructs were prepared by replacing the octamer region from −3305 to −3209 in the same pcDNA3 vector containing the mouse H19 upstream region with a unique *Spe*I site via overlapping PCR. WT and mutant 83 bp sequences corresponding to the deleted region were constructed by ligating three double-strand oligonucleotides containing the appropriate mutations into a *Spe*I linearized vector. (See Table S1 for WT and mutant sequences.)

Luciferase reporter constructs were prepared by inserting either a 352 bp WT or mutant octamer PCR product (−3396 to −3044) or the 83 bp WT or mutant Sox sequence into the *Nhe*I site of the pH19-Luc vector [26]. All PCR-derived constructs were sequenced to ensure fidelity.

Oct4 and Sox2 recombinant protein expression vectors were prepared by cloning *BamH*I – *Sal*I fragments containing the respective cDNAs from pMXS-Oct3/4 and pMXS-Sox2 (Addgene plasmids 13366 and 13367 [31]) into the *BamH*I site of pET-15b (Novagen).

The mutant octamer knock-in vector was prepared by ligating the mutant ICR contained within an *Xba*I fragment from −5.5 to −0.8 kb between the *lox5*11 and *lox*P sites of a previously described targeting vector [9].

### Cell culture and stable transfection

F9, NIH-3T3, C2C12, and HEK293T cells (all from ATCC) were cultured in Dulbecco's modified Eagle's medium (DMEM) supplemented with 10% fetal bovine serum (FBS) and penicillin-streptomycin. F9 cells were cultured on 0.1% gelatin-coated dishes. For ICR demethylation analysis, constructs were treated with *Hha*I and *Hpa*II methyltransferases for partial methylation and *Sss*I methyltransferase for full CpG methylation. Complete methylation was verified by *Hha*I and *Hpa*II restriction digest followed by agarose gel electrophoresis and ethidium bromide staining. Only DNA showing no digestion was used in transfections. Cells were co-transfected with test constructs and either a pcDNA3.1 or pBS-puromycin selection plasmid at a ratio of 5∶1 using either FuGENE 6 [30] or MirusTransIT-LT1 at a lipofectant to plasmid ratio of 3∶1. Cells underwent 10 days of selection with either G418 or puromycin and were then passaged onto 60 mm plates and harvested when confluent.

### Luciferase assays

Transient transfections of F9 cells were performed in six well plates using 3 µL MirusTransIT-LT1 and 0.5 µg each of test construct and pSV-β-gal (Promega). After 48 hours, cells were harvested in Reporter Lysis Buffer (Promega), and β-galactosidase and luciferase activity were assayed as previously described [32]. Relative luciferase expression was calculated after normalizing for β-galactosidase activity. Student's t-test was used to determine statistical significance of at least three independent transfections.

### Lentivirus-mediated Knockdown

Knock-down of Oct4 in F9 cells was achieved using lentiviral particles derived from Sigma-Aldrich MISSION plasmids pLKO.1, (S1 =  Clone ID: NM_013633.1-659s1c1, and S3 =  Clone ID: NM_013633.1-544s1c1). A pLKO.1 vector with a non-targeting shRNA (SCR =  Sigma-Aldrich SHC002) served as a negative control. Lentivirus was produced in 293T cells and used to infect F9 cells on a 6-well plate according to the Addgene pLKO.1 Protocol version 1.0 (December 2006). F9 cells underwent selection with puromycin from 25−48 hrs post-infection and then were passaged onto a 6-well plate. At 72 hrs post-infection, cells were transfected with luciferase vectors and pSV-β-gal and then collected for analysis after 48 hrs. Oct4 knock-down was verified by Western blotting extracts from cells infected in parallel and collected 5 days post-infection using rabbit α-Oct4 [Santa Cruz sc-9081 (1∶500 dilution)] and mouse α-nucleophosmin (NPM) [LabVision ms-1849 (1∶10,000)]. HRP-conjugated human α-rabbit IgG [Sigma-Aldrich A0545 (1∶5,000)] and goat α-mouse IgG [Jackson ImmunoResearch 63656 (1∶8,000)] secondary antibodies. Blots were visualized using Peirce SuperSignal West Pico Chemiluminescent Substrate.

### Southern blotting

To assess ICR methylation, genomic DNA was purified as previously described and digested with *Bgl*II followed by *Hha*I or *Hpa*II methyl-sensitive restriction enzymes [9]. *Msp*I, a methylation insensitive isoschizomer of *Hpa*II, was used as a positive control for complete digestion. Digested DNA was separated by agarose gel electrophoresis followed by blotting to Hybond-XL (Amersham Biosciences). Probes were radiolabeled with the Takara Random Primer DNA Labeling Kit and hybridized to membranes in either Rapid-Hybe Buffer (GE Healthcare) supplemented with 25 µg/ml salmon sperm (SS) DNA at 65°C for 2 hrs or 3% Poly(acrylic acid) pH8.0, 6× SSPE, 1% SDS, 0.1% BSA, 25 ug/ml SS DNA at 65°C overnight. After hybridization membranes were washed in 2× SSC, 0.1% SDS at room temperature for 2 min, twice in 1× SSC, 0.1% SDS at 65°C for 15 min, and once in 0.1× SSC, 0.1% SDS at 65°C for 30 min.

### Electrophoretic mobility shift assays

F9 nuclear extract (NE) was prepared as previously described [33]. Recombinant Oct4 and Sox2 were purified from IPTG induced Rosetta (DE3) pLys competent cells (Novagen) transformed with pET-15b-Oct4 or pET-15b-Sox2 expression vectors and cultured in LB supplemented with ampicillin and chloramphenicol. After lysis with BugBuster Protein Extraction Reagent (Novagen), Oct4 and Sox2 were purified from inclusion bodies by preparative SDS-polyacrylamide gel electrophoresis. Isolation and renaturation of the gel-isolated proteins were performed according to Hager and Burgess [34].

Oligonucleotide probes and competitors used in EMSA are listed in Table S1. For each reaction 25 fmoles of labeled double-stranded probe was incubated for 10 min at room temperature with either F9 NE or recombinant Oct4 and/or Sox2 proteins in a 16 µl reaction buffer containing 10 mM Tris pH8.0, 2 mM MgCl, 100 mM KCl, 1.25 mM DTT, 5% glycerol, 0.0625% BSA, and 19 µg/ml dGdC. Supershifts included an additional 10 min RT incubation with 1 µg of anti-Oct4 (Santa Cruz sc-9081), anti-Sox2 (Santa Cruz sc-20088), or IgG (Santa Cruz sc-2028) as a negative control. Reactions were separated on a 4% 0.25X TBE polyacrylamide gel at room temperature, which was dried before exposing to film. Quantitative analysis of DNA binding data was performed using a Storm 860 Phosphoimager and ImageQuant 5.2 software.

### Chromatin immunoprecipitations (ChIP)

Chromatin from F9 and F9 stable transfectants was prepared and immunoprecipitated using the ChIP-IT Express kit (Active Motif). Chromatin was cross-linked at room temperature with either 1% formaldehyde for 10 min or 2 mM disuccinimidyl glutarate (DSG) 45 min followed by formaldehyde for 10 min (Sox2 endogenous ICR precipitations only) and sonicated using a Vibra Cell (VC 375) ultrasonic processor. Each ChIP reaction contained 25 µg of sheared chromatin precipitated with 6ug anti-Oct4 (Santa Cruz sc-9081), 6 µg anti-Sox2 (3 µg each of Santa Cruz sc-20088 and sc-17320) or 6 µg IgG (Santa Cruz sc-2028 and sc-2027) as a negative control. Precipitated DNA was analyzed using a two-step qRT-PCR reaction in SsoFast EvaGreen Supermix (Bio Rad) on a Bio Rad iCycler. The primers used to amplify the ICR octamer region are: 5′-ATGCAGACCCCACTAAGCAT-3′ and 5′-CGGAGATCATTAGCATCTGA-3′.

### Generation of transgenic mice

ES cell lines and chimeric mice were created by the University of Illinois Transgenic Mouse Facility. CJ7 cells [35] were electroporated with *Sal*I linearized knock-in vector and colonies were selected for G418-resistance. ES clones demonstrating proper targeting by Southern analysis were injected into C57BL/6J (B6) derived blastocysts. Genotyping and confirmation of Cre-mediated deletion of the neo cassette were performed by Southern analysis and PCR of tail DNA (MOPrimer, 5′- ATCATTGGGGCGTTCAGATAATC-3′ and OCR3, 5′-GACAGTGCAAAACAGGTGAA-3′). All experiments involving animal were conducted in accordance with protocols approved by the Animal Care and Use Committee at the University of Illinois at Urbana-Champaign for the purposes of minimizing animal suffering (IACUC Protocol #9250).

### Oocyte and blastocyst isolation

Unfertilized oocytes were collected from the oviducts of superovulated mice approximately 18 h after hCG was administered. The oocytes were incubated in hyaluronidase to dissociate the cumulus cell-oocyte complexes and then visually sorted and inspected to eliminate cumulus cell contamination. Expanded blastocysts were collected from the uteri of superovulated females 84-90 h after hCG injection and mating. All blastocysts were sorted and visually inspected to eliminate contaminating cells and debris.

### Germ cell isolation

Embryos for PGC isolation were obtained from matings between mutant ICR mice and B6;CBA-Tg (Pou5f1-EGFP) [36]. Embryos were sexed and staged according to morphology [37]. Embryonic gonads were separated from the mesonephros, digested with 150 µl of trypsin-EDTA [0.2% trypsin, 1 mM EDTA, 1× phosphate-buffered saline (PBS)] for 15 min at 37°C, washed with DMEM supplemented with 10% fetal bovine serum and manually triturated to a single cell suspension using a 1 ml syringe and 30G needle. GFP-positive cells were isolated by flow cytometry on a BD Biosciences LSR II. PGC purity was verified by assaying for alkaline phosphatase activity using the Vector Blue Phosphatase Substrate Kit III (Vector Laboratories).

### Bisulfite sequencing

Genomic DNA from PGCs, oocytes, and blastocysts was prepared and bisulfite treated using the EZ DNA Methylation-Direct Kit (Zymo Research) according to the manufacturer's instructions. Genomic DNA from neonatal liver and muscle was prepared separately and added directly to the conversion reagent. Primers (Bi3000F and Bi3455R) and PCR conditions for amplification of CTCF site 4 were as previously described [9]. Amplification of CTCF site 2 and the octamer region were performed using the following primer set: Bi1825F, 5′-TTGTAAAGAATTTTTTGTGTGTAAAG-3′ and Bi2310R, 5′-ATACAATTTCAAAATTATTTACAACCC-3′. PCR was performed for 35−40 cycles under the following conditions: 95°C, 30 s; 52°C 40 s; 72°C, 100 s. For oocytes, a secondary PCR with 1−5 µl of the first reaction was performed for 35 cycles with the same parameters. PCR products were gel-purified and cloned following the manufacture's protocol (pGEM-T, Promega). Sequence analysis was performed using BISMA software [38].

### RNA isolation and SNuPE analysis

Tissue was dissected from WT and mutant 4 dpn pups obtained from mating female mice heterozygous for the mutant ICR with males that were homozygous for the *M. casteneus Igf2/H19* locus. RNA was purified using the RNeasy Mini Kit (Qiagen), and all samples were subjected to on-column DNase digestion (RNase-Free DNase Set, Qiagen). Tissue was homogenized for 3 min in RLT buffer using 0.1 mm Zirconia/Silica beads and a Mini-BeadBeater (Biospecs Products). Igf2 and H19 transcripts were amplified using the SuperScript First Strand Synthesis System (Invitrogen), and the following primer sets: Igf2-168F/U, 5′- ACGTGTCTACCTCTCAGGCCGTACT-3′ and Igf2-166R, 5′-GGGTTGTTTAGAGCCAATCAA-3′ [39]; H19-172F, 5′-GCACTAAGTCGATTGCACTGG-3′and H19-172R, 5′-GCCTCAAGCACACGGCCACA-3′ [40]. PCR was performed for 35 cycles under the following conditions: (Igf2) 95°C, 20 s; 53°C 20 s; 72°C, 30 s; and (H19) 95°C, 20 s; 56°C 20 s; 72°C, 20 s. Control reactions lacking RT did not yield a PCR product. SNuPE was performed as previously described [41] using the following primers: Igf2-169R/S, 5′-TCAAATTTGGTTTTTTAGAA-3′ [39]; H19-173R/S, 5′-GGCAGCATTGCCAAAGAGG-3′ [39]. Quantification of SNuPE reactions following electrophoresis was performed using a Storm 860 Phosphoimager and ImageQuant 5.2 software.

## Supporting Information

Figure S1 **Methylation sensitive Southern analysis of WT and mutant ICR transgenes in F9, C2C12 and 3T3 mouse cell lines.** ICR constructs and assays were identical to those described in Fig. 1 except where indicated. All transgenes were either partially (*Hha*I and *Hpa*II) or fully methylated (*Sss*I) prior to transfection. (**A**) Transgenes with mutations in the octamer upstream of CTCF site 1 (shown in Fig. 1A) alone or in combination with CTCF site mutations were stably incorporated in F9 cells. **(B)** WT and mutant ICR transgenes were stably incorporated in C2C12 cells and 3T3 cells **(C)** The fully methylated WT ICR transgene was stably incorporated in F9 cells. **(D)** An ICR transgene containing mutations at both SoxA and SoxC was stably incorporated into F9 cells. See Table S1 for WT and Mutant sequences.
(TIF)

Figure S2 **EMSAs with WT Sox-Oct probes for mouse and human ICR conducted with recombinant Oct4 and Sox2.** **(A)** Quantitative analysis of DNA binding data from Fig. 2E. The amount of probe in each protein-DNA complex is represented as the percentage of total probe (bound and unbound) for each sample. The predicted percentage of Sox2-Oct4 ternary complex was determined by multiplying the fraction of probe bound by Oct4 in the absence of Sox2 and the fraction of probe bound by Sox2 in the absence of Oct4 (EMSA not shown). **(B)** Probes containing conserved Sox-Oct motifs from the Human ICR A2 repeat were incubated with Sox2 and increasing amounts of Oct4. **(C)** Probes containing both mouse Sox-OctA and B or human OctA and Sox-OctB of the Human A2 repeat were incubated with Sox2 and increasing amounts of Oct4. Probe sequences are listed in Table SI.
(TIF)

Figure S3 **Bisulfite analysis of WT and mutant maternally transmitted ICRs from neonatal muscle.** Bisulfite sequencing was performed on DNA isolated from the leg muscle of two different 4 dpn pups (one each for WT and mutant) resulting from reciprocal crosses of mice possessing the WT *cast*. and mutant B6 allele. A polymorphism on the WT *cast*.allele eliminates a single CpG located between the two octamer sites.
(TIF)

Table S1 EMSA oligonucleotides.
(TIF)
