# Supplementary figures and images for: Oct4/Sox2 Binding Sites Contribute to Maintaining Hypomethylation of the Maternal Igf2/H19 Imprinting Control Region
Source: PLoS One. 2013 Dec 6;8(12):e81962. doi: 10.1371/journal.pone.0081962 (PMC3855764; doi:10.1371/journal.pone.0081962)

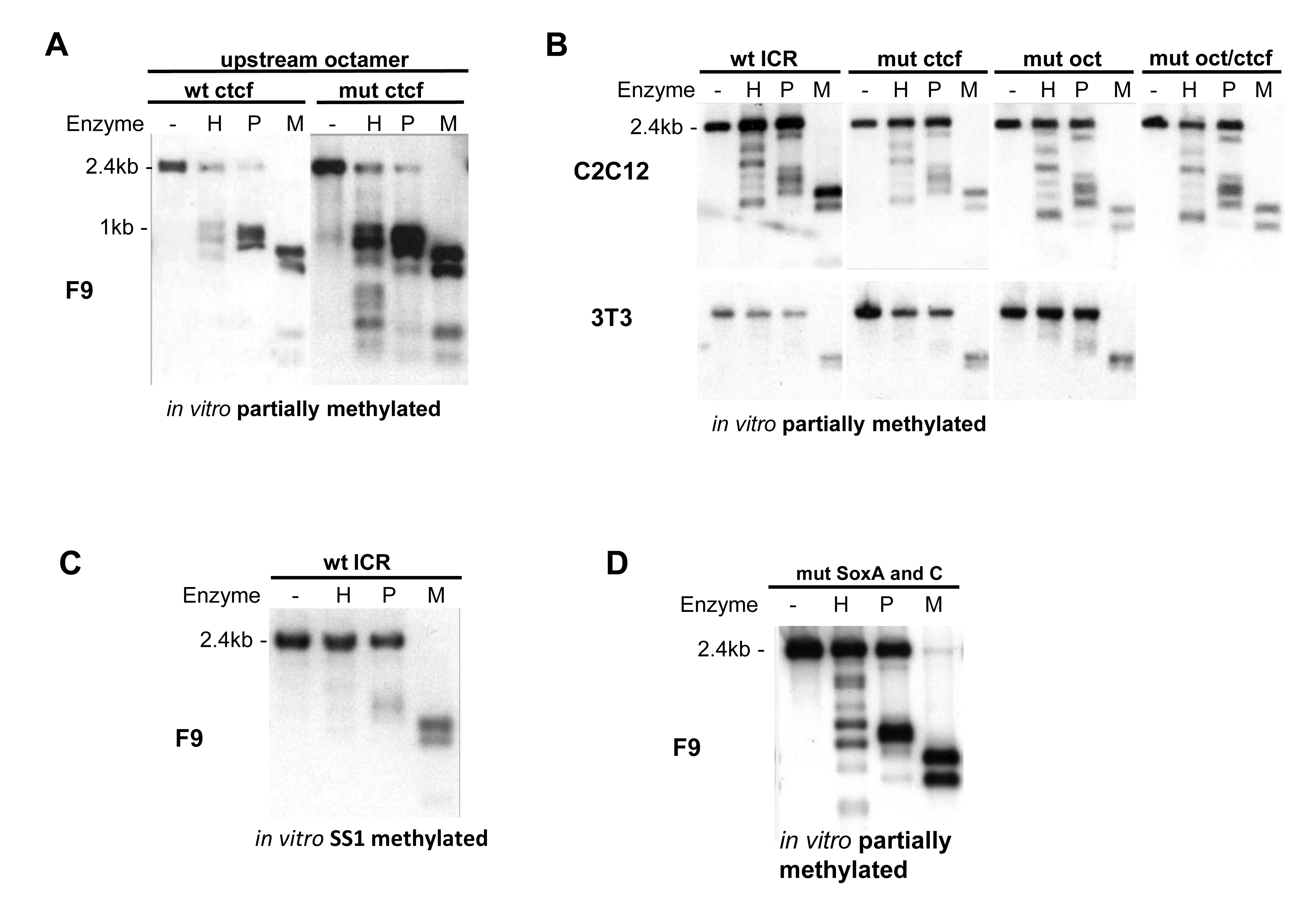

Supplement: Figure S1 — Methylation sensitive Southern analysis of WT and mutant ICR transgenes in F9, C2C12 and 3T3 mouse cell lines. ICR constructs and assays were identical to those described in Fig. 1 except where indicated. All transgenes were either partially (HhaI and HpaII) or fully methylated (SssI) prior to transfection. (A) Transgenes with mutations in the octamer upstream of CTCF site 1 (shown in Fig. 1A) alone or in combination with CTCF site mutations were stably incorporated in F9 cells. (B) WT and mutant ICR transgenes were stably incorporated in C2C12 cells and 3T3 cells (C) The fully methylated WT ICR transgene was stably incorporated in F9 cells. (D) An ICR transgene containing mutations at both SoxA and SoxC was stably incorporated into F9 cells. See Table S1 for WT and Mutant sequences. (TIF) [file pone.0081962.s001.tif]

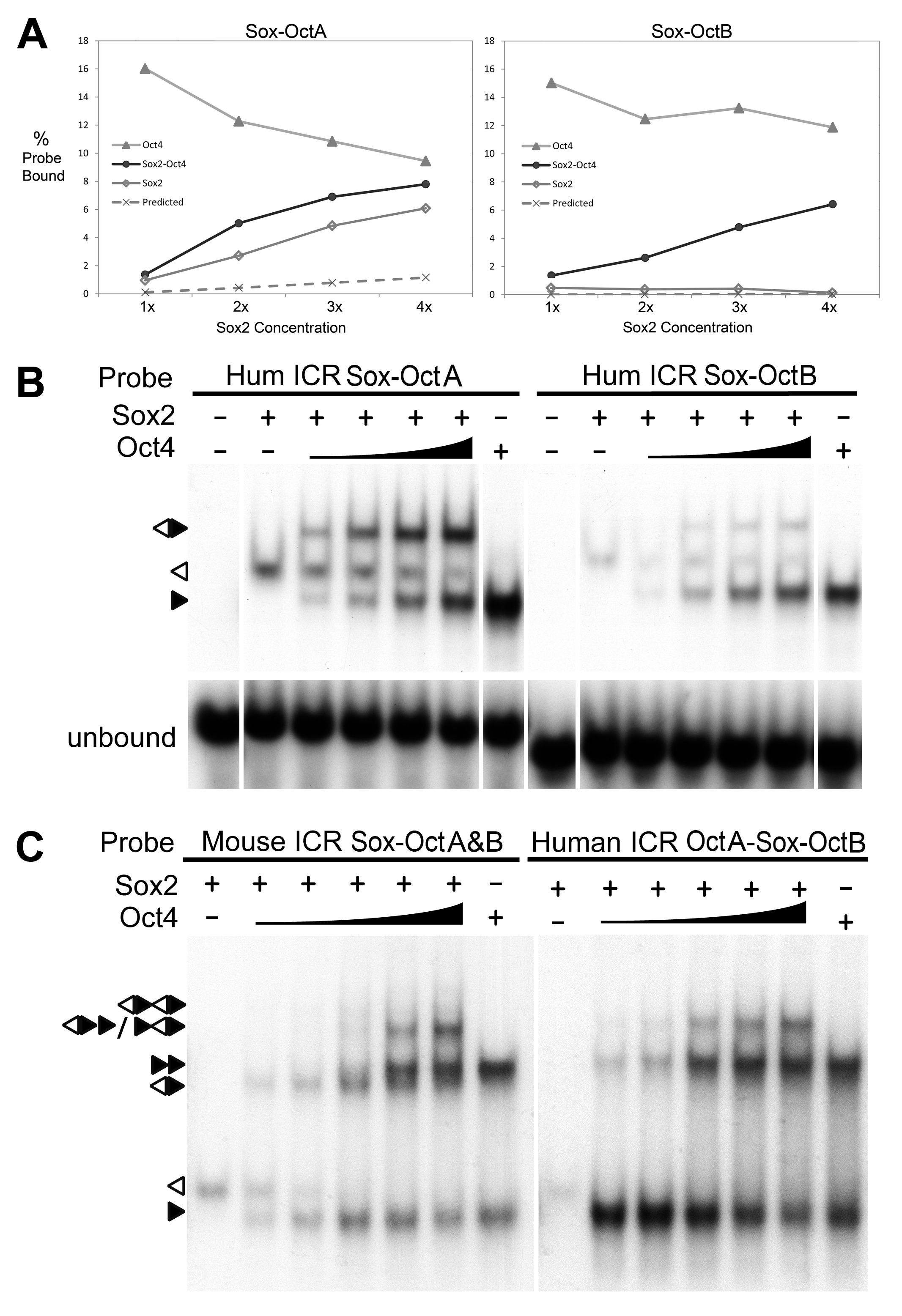

Supplement: Figure S2 — EMSAs with WT Sox-Oct probes for mouse and human ICR conducted with recombinant Oct4 and Sox2. (A) Quantitative analysis of DNA binding data from Fig. 2E. The amount of probe in each protein-DNA complex is represented as the percentage of total probe (bound and unbound) for each sample. The predicted percentage of Sox2-Oct4 ternary complex was determined by multiplying the fraction of probe bound by Oct4 in the absence of Sox2 and the fraction of probe bound by Sox2 in the absence of Oct4 (EMSA not shown). (B) Probes containing conserved Sox-Oct motifs from the Human ICR A2 repeat were incubated with Sox2 and increasing amounts of Oct4. (C) Probes containing both mouse Sox-OctA and B or human OctA and Sox-OctB of the Human A2 repeat were incubated with Sox2 and increasing amounts of Oct4. Probe sequences are listed in Table SI. (TIF) [file pone.0081962.s002.tif]

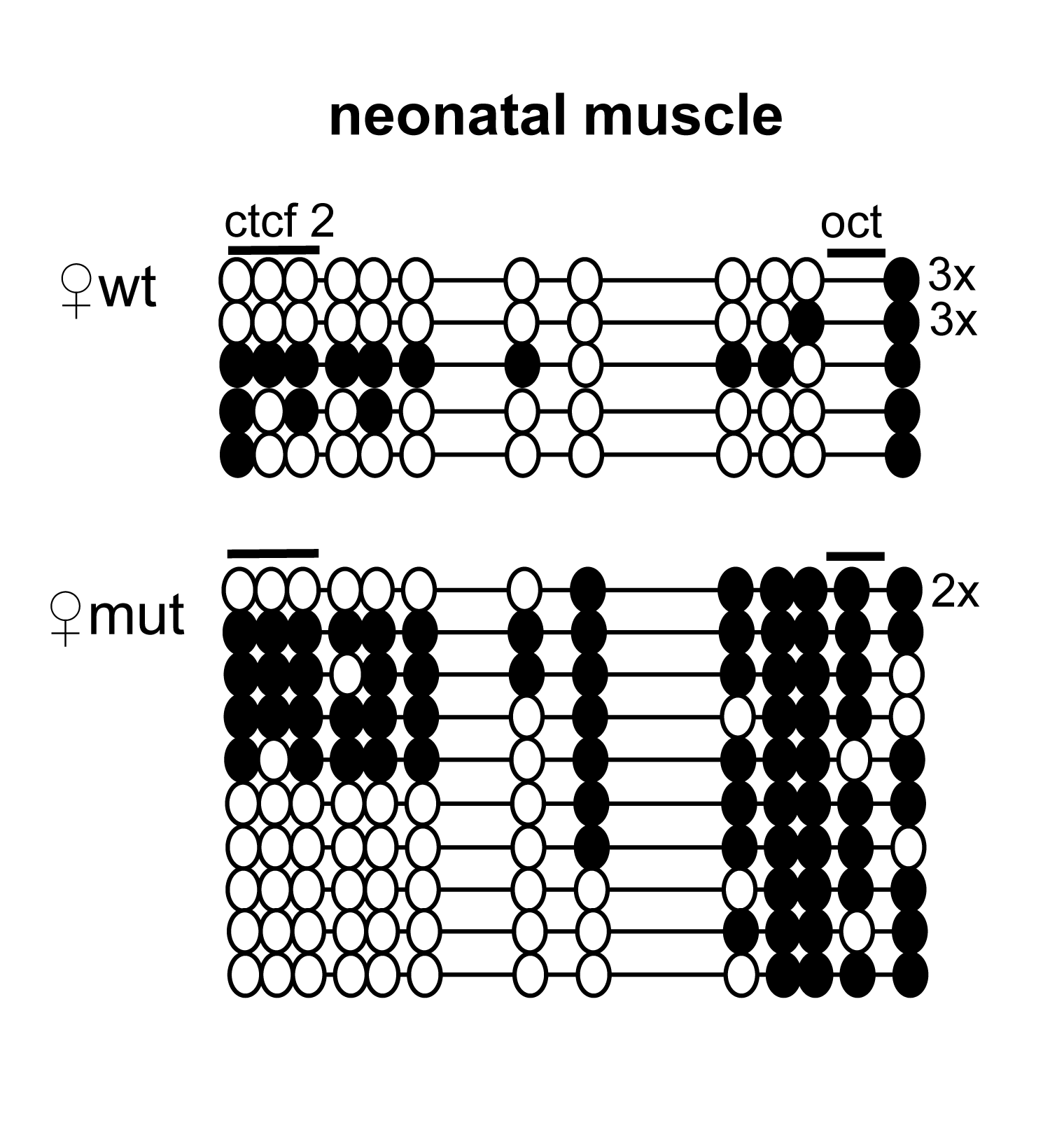

Supplement: Figure S3 — Bisulfite analysis of WT and mutant maternally transmitted ICRs from neonatal muscle. Bisulfite sequencing was performed on DNA isolated from the leg muscle of two different 4 dpn pups (one each for WT and mutant) resulting from reciprocal crosses of mice possessing the WT cast. and mutant B6 allele. A polymorphism on the WT cast.allele eliminates a single CpG located between the two octamer sites. (TIF) [file pone.0081962.s003.tif]

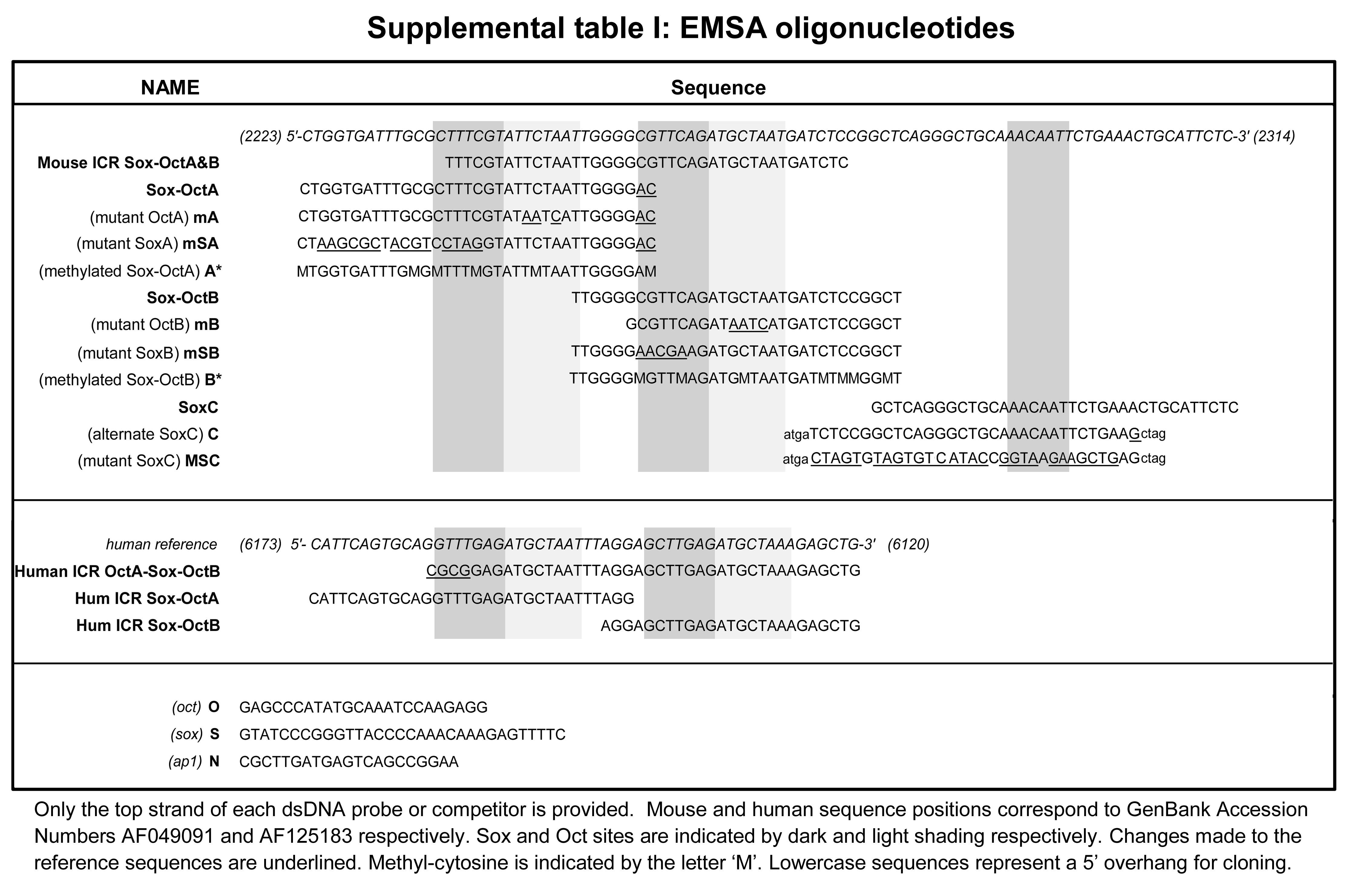

Supplement: Table S1 — EMSA oligonucleotides. (TIF) [file pone.0081962.s004.tif]
